# Better together: Reciprocal activation of quorum sensing circuits enhances bacterial communication

**DOI:** 10.1371/journal.pbio.3003347

**Published:** 2025-09-05

**Authors:** Andrew Frando, Ajai A. Dandekar, Josephine R. Chandler

**Affiliations:** 1 Department of Medicine, University of Washington, Seattle, Washington, United States of America; 2 Department of Molecular Biosciences, University of Kansas, Lawrence, Kansas, United States of America

## Abstract

Many bacteria use quorum sensing to control gene transcription based on population density. This primer explores a recent study in PLOS Biology which found that in Pseudomonas aeruginosa, two quorum-sensing circuits activate each other, enhancing robustness and explaining their redundancy.

## Teaser

Many bacteria use quorum sensing to control gene transcription based on population density. A recent study in *PLOS Biology* found that in *Pseudomonas aeruginosa*, two quorum sensing circuits activate each other, enhancing robustness and explaining their redundancy.

Many bacteria communicate with one another using quorum sensing, which enables bacteria to sense and respond to changes in population density. These systems can activate dozens to hundreds of gene targets, many of which are important for virulence or for optimizing survival strategies in challenging environments. Quorum sensing has received much attention as a potential target for novel anti-virulence therapeutics, synthetic biology, and understanding bacterial interactions more generally.

**Fig 1:**
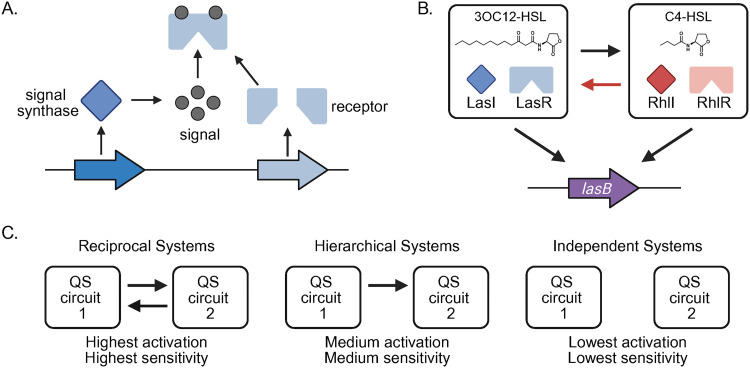
System architecture of quorum-sensing circuits. (A) Quorum sensing in proteobacteria. A signal synthase (blue) produces a diffusible signal (black circles) that binds to a cytoplasmic signal receptor (light blue). When the signal reaches sufficient concentrations, it binds to and activates the receptor, which causes changes in transcription of target genes. (B) The*las* and *rhl*systems of *P. aeruginosa.*The *lasB* gene is activated by both systems. The specific acyl-homoserine lactone signal molecule is pictured for each system. (C) Illustration of circuit architecture: reciprocal, hierarchical, and independent. Indicated below are the relative levels of *lasB* gene activation and sensitivity shown by Thomas and colleagues in experimental and modeling studies.

In proteobacteria, one type of quorum sensing involves a cognate protein pair consisting of a signal-producing synthase and a signal-responsive regulator, in which the regulator often functions as a transcriptional activator [1,2] (Fig 1A). The signals are produced at low levels, diffuse into the environment, and accumulate as population density increases or environmental diffusion decreases. When the signal reaches sufficient concentrations, it binds to and activates its cognate signal receptor to cause changes in gene transcription, and therefore, behavior [3,4]. Some bacteria have a single synthase-receptor pair (referred to as a circuit), but many have multiple circuits that each produce and respond to distinctly different signals. A major question in the field is why some bacteria have multiple quorum-sensing circuits and others only have one. A recent *PLOS Biology* study by Thomas and colleagues [5] addresses this question by leveraging the multiple quorum-sensing systems of a well-studied human pathogen, *Pseudomonas aeruginosa*. Their study investigates how interactions between quorum-sensing circuits affect downstream gene regulation and demonstrates that circuit interactions optimize the overall system’s ability to respond to changing conditions, which may be an advantage of having multiple quorum-sensing circuits.

In *P. aeruginosa,* the *las* and *rhl* quorum-sensing circuits produce and respond to structurally related acyl-homoserine lactone signaling molecules to control distinct but overlapping regulons (Fig 1B). The *las* circuit activates the *rhl* circuit, representing a hierarchical architecture [6]. Thomas and colleagues [5] first asked if there might be additional layers to the architecture of the *P. aeruginosa* quorum-sensing circuits. They used strains with null mutations in one or both systems and bioluminescence reporters of the *las* or *rhl* signal synthase genes to assess circuit activation. They show, consistent with prior findings, that the *las* system activates the *rhl* system, but, importantly, they also show that the *rhl* system reciprocally activates the *las* system. This new finding demonstrates the *las* and *rhl* circuits have a reciprocal interaction rather than a strictly hierarchical one.

Next, they explore the potential advantage of having this kind of arrangement—or, more generally, of having multiple circuits. To do this, they again use transcription reporters and combine their experimental results with mathematical modeling in an elegantly designed, complementary approach. They focus on *lasB*, a gene regulated by both the *las* and *rhl* circuits [7]. First, they experimentally measure *lasB* activation by each circuit alone and by both circuits together. They find that when both circuits are active, *lasB* expression is non-additive and synergistic; that is, it is higher than expected from the sum of their individual effects. This finding indicated that reciprocal interactions between circuits may lead to a more robust activation of downstream gene targets.

Next, they used mathematical modeling to validate their experimental findings. The modeling demonstrated that quorum-sensing circuits with reciprocal interactions—where the *las* and *rhl* systems activate each other—better predicted the experimental data than models assuming hierarchical or independent circuit arrangements. This result confirmed that reciprocal interactions can enhance the robustness of target gene activation. The modeling results also showed that such reciprocal circuits are more sensitive to changes in population density and diffusion than hierarchical or independent circuits (Fig 1C). Together, the experimental and modeling results support the idea that having multiple circuits enables a more dynamic and precise control of gene regulation, making it more sensitive to changes in population density and more robust to changes in diffusion.

The work by Thomas and colleagues reshapes the long-held view of the relationship of the *las* and *rhl* circuits in *P. aeruginosa* and how these two systems regulate their gene targets. Their work also significantly advances our understanding of why bacteria might maintain multiple quorum-sensing circuits and the complex gene regulation that arises from such arrangements. The discovery that reciprocally activating circuits can synergistically activate gene targets opens a new avenue for examining the advantages of multilayered quorum-sensing circuitry. For example, reciprocal circuit activation may increase metabolic prudence by mitigating the fitness costs of producing quorum-sensing-regulated goods [8]. Although the authors highlight synergy as a clear benefit of having two circuits, alternative explanations, such as functional redundancy to safeguard critical gene regulation if one system fails [9], remain plausible and require further investigation.

The authors also establish a novel framework that is useful for understanding the interactions of quorum-sensing circuits in other bacteria and also for revealing principles of biological circuit design more generally. Many biological systems use feedback and cross-regulation [10], and this study provides a scaffold for understanding how circuit architecture facilitates precise control and adaptability. One limitation of the study is that the mechanism of reciprocal activation of the *las* and *rhl* systems remains unexplored. For example, activation could be via transcriptional effects on the signal synthase or receptor gene, or via signal-dependent crosstalk between the systems, where the receptor directly binds and responds to the other system’s signal. Further insight into the mechanism of activation could inform understanding of the advantages of different types of circuit interactions. Likewise, the authors focused on a single gene promoter activated by both the *las* and *rhl* circuits (*lasB*), and it would be interesting to know if their observations extend to other genes, particularly those regulated by only one of the two circuits. Future studies might expand this model to other biological systems or make predictions to optimize circuits in synthetic biology applications. The framework can also be used to ask if there is variation in other quorum-sensing-regulated genes or under diverse environmental conditions, helping to clarify additional factors that shape bacterial communication and behavior. Despite decades of research on these systems in *P. aeruginosa*, as Thomas and colleagues show, there is still much to uncover.
